# Diquat poisoning, maybe another cause of osmotic demyelination syndrome: case report and literature review

**DOI:** 10.3389/fmed.2025.1465003

**Published:** 2025-06-24

**Authors:** Wenfu Li, Hui Zhou

**Affiliations:** ^1^Department of Radiology, Affiliated Hospital of Zunyi Medical University, Medical Imaging Center of Guizhou Province, Zunyi, China; ^2^Department of Graduate School, Zunyi Medical University, Zunyi, China

**Keywords:** diquat, toxic encephalopathy, osmotic demyelination syndrome, magnetic resonance imaging, case report

## Abstract

Diquat (1,1′-ethylene-2,2′-bipyridine), a non-selective herbicide with significant human toxicity, is increasingly used as a substitute for paraquat in weed management practices in China. Diquat intoxication is typified by multiple organ dysfunction syndrome (MODS), predominantly manifesting as acute renal and hepatic injury, and frequently resulting in central nervous system (CNS) impairment with a poor prognosis in severe instances. Despite the rising incidence of diquat poisoning, the imaging characteristics of diquat-induced toxic encephalopathy remain inadequately documented in the literature. In this report, we present a distinctive case involving a female pediatric patient who exhibited MODS affecting the neurologic, renal, hepatic, cardiac, and gastrointestinal systems, in conjunction with rhabdomyolysis. Magnetic resonance imaging (MRI) revealed multiple abnormal signals in the pons, bilateral brachium pontis, thalamus, caudate nucleus, putamen, posterior part of the external capsule, and posterior limb of the right internal capsule. These findings are consistent with the imaging characteristics of osmotic demyelination syndrome (ODS), which is under-recognized but important. After comprehensive systemic treatment, the patient was discharged on the 30th day post-admission.

## Introduction

ODS is an acute non-inflammatory demyelinating condition frequently attributed to the rapid correction of hyponatremia (1, 2). The risk of developing ODS is exacerbated by factors such as post-liver transplantation, chronic alcoholism, malnutrition, hypokalemia, and hypophosphatemia. In the past, poisoning has not been recognized as an etiological factor of ODS, although an increasing number of studies have indicated that the MRI characteristics of diquat toxic encephalopathy align with those observed in ODS (3, 4). ODS includes central pontine myelinolysis (CPM) and extrapontine myelinolysis (EPM), and when the pons is involved, the trident or piglet signs are characteristic MRI manifestations of ODS (5, 6). Toxic encephalopathy predominantly induces alterations in cerebral white matter fibers and subcortical gray matter nuclei, potentially involving the cerebellopontine nucleus. However, imaging findings consistent with ODS are infrequent. Consequently, this study aims to elucidate the MRI manifestations of diquat-induced toxic encephalopathy, which may present distinctive yet under recognized features. Crucially, these imaging characteristics can assist clinicians and radiologists in formulating diagnostic hypotheses in instances where the history of toxic exposure is ambiguous, thereby facilitating accurate diagnosis and prompt, effective treatment.

## Case description

A 13-year-old female patient was admitted to our hospital with anuria and bilateral lower extremity edema lasting for one day. One week prior, the patient experienced abdominal pain and headache without a clear precipitating cause, not associated with vomiting or diarrhea, and was managed with oral medication at a nearby county hospital. Three days before admission, the patient’s symptoms of headache and abdominal pain worsened and were accompanied by vomiting, increased frequency of bowel movements, and fever. The patient was admitted to the local county hospital of traditional Chinese medicine one day prior, presenting with unresolved headache and abdominal pain, as well as the onset of anuria and bilateral lower extremity swelling. Due to the unknown etiology and rapid progression of her condition, she was transferred to our hospital on January 10, 2024, for comprehensive evaluation and management. The patient’s mother has a history of chronic hepatitis B virus (HBV) infection, suggesting that the patient may have acquired HBV through vertical transmission. There is no family history of other genetic or neurological diseases. The patient was previously diagnosed as an HBV carrier, with liver function consistently maintained within the normal range, and did not receive systematic antiviral treatment. Prior to admission, the patient had no significant medical history. Upon admission, the patient was conscious and denied any history of toxic exposure. Vital signs indicated a temperature of 36.8°C, a pulse of 95 beats/min, respiration rate of 20 beats/min, and blood pressure of 118/68 mmHg. Physical examination revealed abdominal diffuse tenderness and nonpitting edema in both lower extremities. Laboratory test results are detailed in Table 1, with electrocardiographic (ECG) findings suggesting T-wave changes. Non-contrast computer tomography (CT) scans of the brain, chest, and abdomen showed normal results.

**Table 1:** Biochemical blood test results.

Test Time	BUN mmol/l	Cr μmol/l	AST U/L	ALT U/L	CK U/L	CK-MB U/L	Mb ng/mL	hsTnT ng/L	NTProBNP Pg/mL	Na^+^ mmol/L	K^+^ mmol/L	WBC	ALB	Hb
Normal values	2.8–7.2*	30–90*	13–35*	7–40*	26–140*	0–24*	25–58*	<14*	<125*	137–147*	3.5–5.3*	3.5–9.5*	40–55*	115–150*
Day1	20.4	584	378	375	5,660	130	>3,000	131.6	1,787	125.2	4.59	32.77	33.1	157.0
Day3	9.7	252	129	200	2,777	62	–	–	–	132.4	3.96	32.93	22.3	157.0
Day4	11.2	215	83	140	1,732	38	–	–	–	137.1	4.38	26.16	21.2	149.0
Day6	–	–	–	–	–	–	>3,000	77.94	143	–	–	–	–	–
Day8	14,2	298	84	113	835	18	–	–	–	128,6	4.52	14.20	23.9	90.0
Day11	12.2	306	48	65	212	7	–	–	–	130.9	4.68	20.80	27.4	78.0
Day13	12	341	39	50	301	11	–	–	–	–	–	–	30.8	–
Day15	–	–	–	–	–	–	393	116.50	–	–	–	13.83	–	83.0
Day16	16.1	324	22	23	228	12	–	–	–	137.4	3.37	–	33.7	–
Day20	5.4	73	21	19	120	20	73	91.29	–	142	3.33	–	43.4	–
Day22	8.5	56	26	29	66	13	26	55.56	280	143.5	3.71	7.64	49.2	102.0
Day27	–	–	50	100	22	6	21	48.54	–	–	–	9.65	46.8	111.0

BUN, blood urea nitrogen; Cr, creatinine; AST, aspartate aminotransferase; ALT, alanine aminotransferase; CK, creatine kinase; CK-MB, creatine kinase muscle and brain isoenzyme; Mb, myoglobin; hsTnT, hypersensitive troponin T; NTProBNP, N-terminal prohormone of brain natriuretic peptide; Na^+^, sodium; K^+^, Potassium; WBC, white blood cell; ALB, Albumin; Hb, Hemoglobin. The asterisk (*) indicates the normal reference range for each indicator.

**Figure 1:**
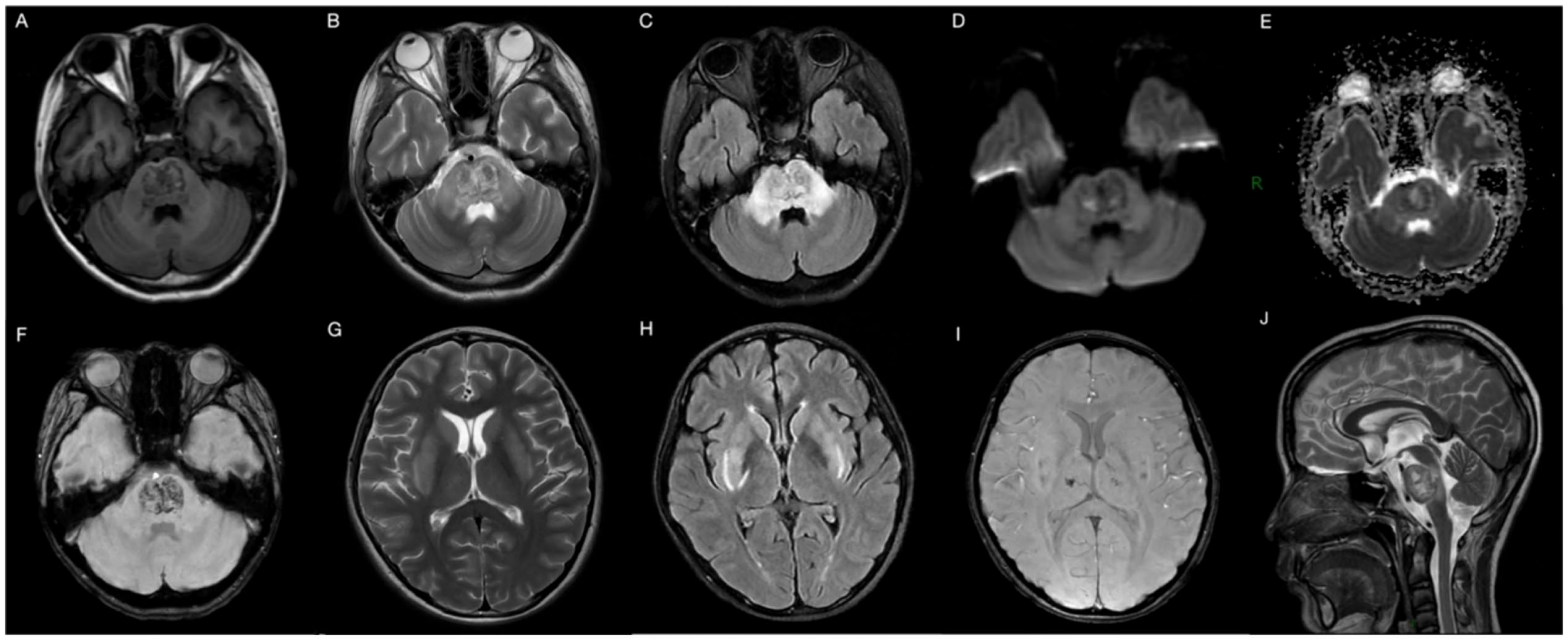
Brain MRI 29 days after ingestion showing abnormal heterogeneous intensities in the pons, bilateral brachium pontis, thalamus, caudate nucleus, putamen, posterior part of the external capsule, and posterior limb of the right internal capsule on T_1_WI **(A)**, T_2_WI **(B,G,J)**, T_2_-FLAIR **(C,H)**, DWI **(D)**, ADC-map **(E)**, and SWI **(F,I)**, in accordance with ODS. T_1_WI, T_1_-weighted imaging; T_2_WI, T_2_-weighted imaging; T_2_-FLAIR, T_2_-fluid-attenuated inversion recovery; DWI, diffusion-weighted imaging; ADC, apparent diffusion coefficient; SWI, susceptibility weighted imaging.

**Figure 2:**
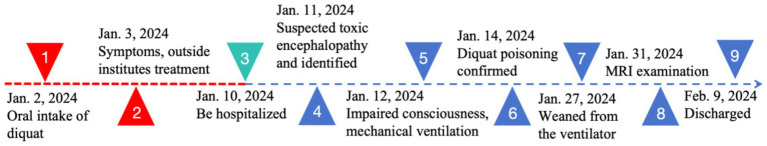
Patient’s treatment timeline.

Following admission, the patient received prompt administration of continuous renal replacement therapy, anti-infection treatment, and symptomatic supportive therapy. On the second day of hospitalization, the patient exhibited behavioral symptoms, including heightened speech and disorientation. Physical examination revealed that the patient’s lips exhibited a burning-like appearance, accompanied by significant congestion, fissures, and scab formation. Experienced attending physicians hypothesized poisoning as the underlying cause. Upon further detailed history-taking, it was revealed that the patient had orally ingested approximately 50 mL of diquat (20 g/100 mL) 9 days prior. Subsequently, the blood and dialysate samples were transferred to an external medical facility for analysis of toxic substance composition, revealing diquat concentrations of 12 ng/mL in the blood and 80 ng/mL in the dialysate. The patient exhibited symptoms of impaired consciousness, rapid respiration, elevated heart rate, and unstable oxygen saturation 3 days post-admission, necessitating immediate initiation of invasive ventilator-assisted ventilation. This intervention continued until day 17, at which point the patient’s condition began to improve. On the 21st day post-admission, a brain MRI examination was performed using a 3.0 T magnetic resonance scanner (Discovery MR750w, General Electric, Milwaukee, WI, USA) with a 32-channel dedicated head coil. Specific scan sequence parameters are shown in Table 2. Magnetic resonance imaging revealed multiple abnormal signals in various regions, including the pons, bilateral brachium pontis, thalamus, caudate nucleus, putamen, posterior part of the external capsule, and posterior limb of the right internal capsule. These regions exhibited hypointense signals on T_1_-weighted imaging (T_1_WI), hyperintense signals on T_2_-weighted imaging (T_2_WI) and T_2_-fluid-attenuated inversion recovery (T_2_-FLAIR), and hypointense signals on susceptibility-weighted imaging (SWI). Partial lesions in the pons exhibited hyperintense signals on diffusion-weighted imaging (DWI) and reduced signals on the apparent diffusion coefficient (ADC) map (Figure 1). The central region of the pons exhibited severe damage with hyperintense signals on T_2_WI, while the corticospinal and corticobulbar tracts were relatively preserved, showing hypointense signals, forming the characteristic “piglet sign” (Figure 1B). These findings were entirely consistent with the diagnosis of ODS. Following systemic treatment, the patient was discharged on the 30th day post-admission. At the time of discharge, the patient was conscious, able to understand medical instructions and produce simple sounds, but unable to answer questions. She was also unable to eat or walk independently and occasionally coughed while drinking. After multiple rehabilitation sessions at a large tertiary hospital in Guangzhou, Guangdong Province, China, as of the time of manuscript preparation (4 months post-discharge), the patient has largely recovered to a level indistinguishable from a healthy individual, although her personality has become extremely irritable compared to her pre-hospitalization state. The timeline of treatment can be seen in Figure 2.

**Table 2:** The specific sequence and parameters of the patient’s brain MRI.

Sequence	TR (ms)	TE (ms)	Flip angle (°)	Matrix size	FOV (mm)	Slice thickness (mm)	Slice spacing (mm)
T_1_WI	2,250	24	111	320 × 224	240 × 240	5	1.5
T_2_WI	4,675	90	142	416 × 416	240 × 240	5	1.5
T_2_-FLAIR	7,000	140	160	320 × 224	240 × 240	5	1.5
DWI	4,342	Minimum	–	128 × 160	240 × 240	5	1.5
SWI	Minimum	Out of phase	15	384 × 320	240 × 192	2.6	0

TR, repetition time; TE, Echo Time; FOV, Field of View; T_1_WI, T_1_-weighted imaging; T_2_WI, T_2_-weighted imaging; T_2_-FLAIR, T_2_-fluid-attenuated inversion recovery; DWI, diffusion-weighted imaging; SWI, susceptibility weighted imaging.

## Literature review

**Figure 3:**
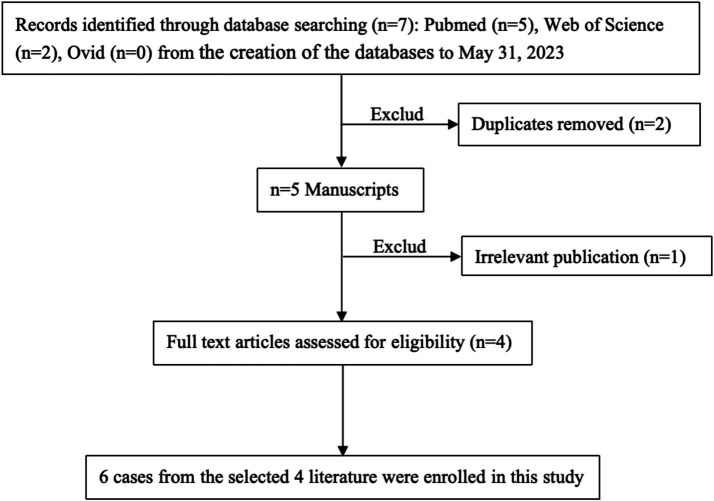
The literature screening process flow chart for diquat toxic encephalopathy with MRI manifestations.

To understand the imaging manifestations of diquat toxic encephalopathy, we performed a literature review of all English-language case reports of diquat poisoning that contained MRI manifestations in PubMed, Web of Science, and Ovid databases, dated from the creation of the databases to May 31, 2024. Keywords were used for the search, which included “diquat,” “diquat dibromide,” “magnetic resonance imaging,” “magnetic resonance image,” and “MRI.” The flowchart illustrating the literature screening process is depicted in Figure 3, with a total of four articles encompassing six cases included in the analysis. Each case was meticulously documented, including details such as the first author and country of authorship, year of publication, patient’s sex and age, clinical presentation, timing of MRI performance post-intoxication, lesion site, prognosis, and duration of follow-up (Table 3).

**Table 3:** The cases of diquat poisoning resulting in toxic encephalopathy with MRI manifestations from the literature review.

Case (No.)	References/year/country	Sex	Age (yrs)	Presentation	Location of the lesions on MRI	TFPME	Prognosis	Follow-up
1	Sechi et al. (7)/1992/Italy	M	72	Severe parkinsonian syndrome	Caudate nuclei, putamen and cerebral white matter near the ventricular wall	4mo	NA	NA
2	Xing et al. (4)/2020/China	M	21	Nausea and vomiting	Pons	11ds	Died of MODS	15ds
3	Yu et al. (3)/2022/China	M	20	Anuric and pharyngeal hyperemia	Brainstem, particularly in the pons, bilateral caudate nucleus, putamen, and capsula externa	18ds	Dystaxia and difficulty in walking	3mo
		M	20	Oliguria, agitation, convulsions, and respiratory failure	Pons, bilateral brachium pontis, and pedunculus cerebr and corona radiata	13ds	Died of cardiac arrest	18ds
		M	31	Nausea and vomiting	medulla oblongata, pons, midbrain, cerebellum, left thalamus, surrounding posterior horn of the lateral ventricle, bilateral brachium pontis, and pedunculus cerebri	4ds	Symptoms were almost completely relieved	57ds
4	Dai et al. (16)/2023/China	M	25	Renal function deteriorated	Splenium of the corpus callosum	9ds	Renal function had improved	19ds
5	PC	F	13	Abdominal pain, anuric, headache and vomiting	Pons, bilateral brachium pontis, thalamus, caudate nucleus, putamen, posterior part of the external capsule, and posterior limb of the right internal capsule	29ds	No discomfort except for a personality change	4mo

PC, present case; F, female; M, male; MRI, magnetic resonance imaging; yrs, years; mo, months; ds, days; MODS, multiple organ dysfunction syndrome; NA, not available; TFPME, Time from poisoning to MRI examination.

Based on the literature, only six cases of diquat toxic encephalopathy, as described in four articles, have been reported to include MRI features. The findings indicated that prior cases exclusively involved males aged between 20 and 72 years, whereas our case represented the youngest individual and the sole female. The patient’s clinical presentation primarily stemmed from gastrointestinal and genitourinary dysfunctions, including symptoms such as nausea, vomiting, renal dysfunction, and anuria. Brain lesions may manifest as either singular or multiple, with the latter typically exhibiting symmetrical distribution. Predominant anatomical locations for lesion involvement include the brainstem, specifically the pons, as well as the bilateral basal ganglia and thalamus. Significantly, 71% (5/7, including our case) of the cases had brain MRI manifestations consistent with ODS, given its specific imaging features. In this cohort, the shortest interval between diquat ingestion and MRI evaluation was 4 days. Unfortunately, two patients died of MODS and cardiac arrest, respectively, while three patients experienced differing degrees of neurological deficits post-discharge, suggesting a poor prognosis for this disease.

## Discussion and conclusion

Diquat and paraquat are both non-selective bipyridine herbicides with similar structures. Following the prohibition of paraquat in numerous countries globally in recent years, the utilization and incidents of intoxication involving diquat have been on the rise as a substitute. Diquat can be absorbed through multiple pathways, including ingestion, inhalation, and mucous membranes, and in rare cases, through intramuscular, subcutaneous, and vaginal injections (7, 8). Gastrointestinal ingestion is the most common route of diquat poisoning, and most of them (approximately 90–95%) are excreted in the feces within 24 h, with only about 2% being absorbed into the blood system for distribution throughout the body (9). Following absorption, the kidneys and liver are the organs most commonly affected, with peak concentrations reached within 2 h leading to various toxic effects on multiple organs and tissues (10). Unlike paraquat poisoning, CNS damage from diquat poisoning is more common and severe, while alveolar epithelial injury is typically mild. The mechanisms of toxicity in diquat toxic encephalopathy are complex and remain unclear, generally thought to be associated with cascade reactions and dopaminergic neuron damage induced by oxidative stress, resulting in nerve cell and neuronal axonal degeneration, and ODS (3, 9, 11, 12). Bonneh-Barkay et al. (11) demonstrated through *in vitro* experiments that diquat can cause severe damage to dopaminergic neurons and a significant reduction in dopamine uptake. During hospitalization, our patient exhibited behavioral abnormalities, such as heightened speech and disorientation, as well as personality changes, including irritability, which are highly consistent with the clinical manifestations of dopaminergic neuron dysfunction. Furthermore, previous literature has reported that patients with diquat poisoning develop severe parkinsonian symptoms (7). Although we currently lack direct objective evidence, based on the literature support and existing clinical evidence, we believe that the hypothesis of “dopaminergic neuron damage” is reasonable. In the past, most studies focused on hepatic and renal injuries, while MRI features of cases with CNS involvement were rarely reported.

The clinical manifestations of diquat poisoning are diverse and non-specific. Oral intake of large quantities (200–300 mL) of diquat will lead to extensive ulcers and even bleeding in the digestive tract (13). Gastrointestinal symptoms are the most prominent early clinical manifestations. Corrosive damage includes burning pain in the mouth, ulcers, mucosal edema, esophageal damage, nausea, vomiting, abdominal pain, and diarrhea. However, abdominal pain caused by diquat poisoning may be related to gastrointestinal lesions (e.g., functional disturbances, mucosal inflammation, or ulcers) or other non-specific factors (e.g., electrolyte imbalances or systemic inflammatory responses). Additionally, CT scans have limited sensitivity for early or functional lesions. Therefore, even if abdominal pain is the earliest and most prominent symptom, non-contrast abdominal CT scans may still yield negative results, as demonstrated in our case. To more accurately assess gastrointestinal pathology, we recommend further imaging studies (e.g., contrast-enhanced CT or MRI) or endoscopic examinations if clinical symptoms persist or worsen. Following absorption, the kidney serves as the primary excretory organ for diquat and is also the primary target organ of injury (14). The severity of renal damage can range from simple proteinuria to acute renal failure. Liver, hematologic, and respiratory injuries can also occur in diquat poisoning, and it is worth noting that when lung injuries are severe, further identification of the toxicant should be performed. Diquat has a poisonous effect on central nervous cells (9). Therefore, CNS symptoms are common, including dizziness, drowsiness, convulsions, coma, excitement, restlessness, and disorientation. In our review, the most frequent symptoms in patients were caused by gastrointestinal and renal damage, and CNS-related symptoms were also common but not prominent at the time of admission, except for one patient who presented with severe Parkinsonian syndrome. In addition, severe diquat poisoning can cause rhabdomyolysis, which may aggravate kidney injury and induce myocardial damage (15).

Electrocardiographic T-wave changes can be either physiological or pathological. In patients with diquat poisoning, electrolyte disturbances, myocardial injury, rhabdomyolysis, and systemic inflammatory responses may all contribute to T-wave changes. However, there are few reported studies on electrocardiographic changes in patients with diquat poisoning. Yu et al. (15) reported a case of an 18-year-old female who developed sinus tachycardia (heart rate of 165 beats per minute) after ingesting approximately 200 mL of diquat (20 g/100 mL) and died of cardiac arrest 29 h after ingestion. In contrast, our patient was fortunate. Although her electrocardiogram showed T-wave changes at admission, after continuous electrocardiographic monitoring, electrolyte correction, supportive care, control of systemic inflammatory response, and management of potential myocardial injury and rhabdomyolysis, a follow-up electrocardiogram on the 20th day of hospitalization revealed a return to normal.

Imaging such as CT and MRI play an important role in the diagnosis and differential diagnosis of diquat toxic encephalopathy, especially MRI has great advantages due to its radiation-free, high soft-tissue resolution and multiparametric imaging features. Therefore, in this article, we focused on its MRI characteristics. Lesions in the brain can be single (16) or multiple (7), and multiple lesions are often symmetrically distributed. Xing et al. (4) reported the case of a 21-year-old male who ingested 100 mL of diquat (20 g/100 mL) and confirmed as CPM by MRI. In 2022, Yu et al. (3) reported three cases of diquat poisoning with MRI features consistent with ODS. In our case, MRI revealed multiple symmetrical intracranial lesions involving the pons and basal ganglia regions and thalamus, with the pons lesions showing characteristic piglet signs on T_2_WI and T_2_-FLAIR, a specific MRI manifestation of ODS (17). Diquat poisoning can also lead to basal ganglia hemorrhage (18), pontine hemorrhage or infarction (19). As an advanced MRI sequence, DWI is useful for cytotoxic edema, where restricted diffusion suggests the presence of acute infarction. SWI is sensitive to detecting paramagnetic (e.g., hemosiderin, deoxyhemoglobin) and antimagnetic substances (e.g., bone minerals, dystrophic calcification). Thus, SWI can be sensitive to detect the hyperintensity presented by microbleeds, as in our case. Based on these results, for patients with neurologic symptoms after diquat poisoning, we recommend a multimodal MRI examination including high-level neurofunctional imaging (e.g., DWI, SWI, etc.), which may be meaningful for the understanding of underlying pathophysiologic mechanisms of diquat toxic encephalopathy and for patient prognosis determination.

Currently, there is no specific antidote for diquat, and when the CNS is involved, there is a high rate of disability and mortality. Fortunately, unlike paraquat, diquat does not accumulate in the lungs and has a better prognosis after intensive care treatment. The clinical treatment protocol is directed at reducing absorption and/or increasing elimination (20). When skin or eyes are exposed to a solution of diquat, remove all the contaminated clothing, and repeated rinsing with water is the first preventive measure. Gastrointestinal tract ingested diquat will not be absorbed quickly, therefore, activated charcoal adsorption, gastric lavage, and catheterization are usually used as early treatment options (18, 21). Measures such as diuresis, hemodialysis, and hemoperfusion help eliminate diquat from the circulation, although they do not remove clinically and toxicologically significant quantities of the herbicide (20).

Overall, acute chemical poisoning is a significant contributor to unplanned hospitalization. Uncertainty history of toxic exposure can lead to delays in treatment, potentially resulting in severe outcomes such as permanent disability or death. Diquat poisoning may be another cause of ODS, although the specific mechanism remains unclear. However, the extant evidence is limited, and further studies that cover more cases are required.

Our case is unique because the patient, a left-behind child, did not disclose her diquat ingestion to her guardian or clinician, delaying optimal treatment. Fortunately, after systematic intervention, she only experienced a personality change. MRI is vital for diagnosing diquat toxic encephalopathy but can be overlooked and misdiagnosed. Thus, if the patient’s condition permits, a timely cranial MRI is essential during clinical treatment.

## Patient perspective

The incidence of diaquat toxic encephalopathy is on the rise, yet its imaging characteristics are inadequately documented and acknowledged. Imaging plays a crucial role in the diagnosis and differentiation of CNS disease. Our review indicates that distinctive intracranial imaging presentations may manifest as soon as 4 days following diquat exposure. Consequently, prompt MRI is imperative in instances of suspected diquat poisoning accompanied by CNS symptoms.

In this particular instance, the patient, a left-behind children, initially withheld information regarding a potential diquat poisoning history, resulting in delayed treatment outside of the hospital setting. Fortunately, following comprehensive care at the Affiliated Hospital of Zunyi Medical University, the patient experienced a successful recovery, ultimately providing solace to her family and fostering a profound gratitude for our healthcare team’s specialized expertise and exceptional care.

## Data Availability

The datasets presented in this article are not readily available because of ethical and privacy restrictions. Requests to access the datasets should be directed to the corresponding author.
